# Modified Stoppa Approach for ORIF of a Paediatric Transverse Acetabular Fracture: Case Report and Systematic Review of Internal Fixation in Children

**DOI:** 10.3390/children13020166

**Published:** 2026-01-24

**Authors:** Massimo Berdini, Roberto Procaccini, Donato Carola, Mario Marinelli, Antonio Gigante

**Affiliations:** Department of Clinical and Molecular Sciences, Clinica Ortopedica dell’Adulto e Pediatrica, Università Politecnica delle Marche, 60126 Ancona, Italy; roberto.procaccini@sanita.marche.it (R.P.); donato.carola@sanita.marche.it (D.C.); mario.marinelli@ospedaliriuniti.marche.it (M.M.); a.p.gigante@staff.univpm.it (A.G.)

**Keywords:** pediatric acetabular fracture, pelvic trauma, triradiate cartilage, open reduction and internal fixation, modified Stoppa approach, infrapectineal plate, pelvic ring injuries, case report, systematic review

## Abstract

**Highlights:**

**What are the main findings?**

- An 11-year-old with a transverse acetabular fracture involving disruption of the triradiate cartilage was successfully treated with ORIF via a modified Stoppa approach, achieving excellent six-month functional recovery (return to daily activities and sport).
- A systematic review of 16 studies of the literature shows that paediatric acetabular fractures treated with ORIF generally have good-to-excellent outcomes when the criteria for surgical treatment are met, and when anatomical reduction and stable fixation are obtained.

**What are the implications of the main findings?**

- The modified Stoppa approach—commonly used in adults—appears to be a safe, effective option in selected skeletally immature patients with fractures involving the anterior column/quadrilateral surface, even when the triradiate cartilage is affected.
- Evidence in the literature is limited, and heterogeneous, careful case selection and timely anatomical reduction are crucial, and current evidence remains insufficient to make firm, general recommendations on the optimal paediatric surgical approach.

**Abstract:**

Background: Paediatric pelvic and acetabular fractures are rare and usually the consequence of high-energy trauma, often associated with life-threatening injuries. The majority are managed non-operatively; however, open reduction and internal fixation (ORIF) is indicated in selected, complex, or displaced, acetabular fractures. The modified Stoppa approach is well established in adults, but has been rarely reported in skeletally immature patients, and evidence guiding surgical approach and fixation in children remains limited. Methods: We report the case of an 11-year-old girl who sustained a transverse acetabular fracture following a high-energy trauma. The fracture was treated with ORIF through a modified Stoppa approach. We also performed a systematic review of the literature, focusing on ORIF of acetabular fractures in children. Results: In our patient, ORIF of the acetabular fracture was performed, achieving an anatomical reduction, 10 days after initial damage-control fixation of a concomitant open tibial plateau fracture. Postoperative management consisted of four weeks of non-weight bearing, followed by progressive weight bearing. At six months, she had returned to full daily activities and sports. The review of the literature identified 16 studies (retrospective series and case reports) describing paediatric acetabular fractures treated with ORIF using plates, screws, or flexible nails. In the literature, good to excellent clinical and radiographic outcomes were reported when anatomical reduction and stable fixation were achieved, although growth disturbance and avascular necrosis were described, particularly in cases with delayed reduction or severe triradiate cartilage injury. Conclusions: Our case illustrates the technical feasibility of the modified Stoppa approach in a skeletally immature patient with a complex acetabular fracture, with excellent mid-term outcome. Although it is not contraindicated in paediatric patients, it should be reserved for treating this type of complex fracture. The available literature supports that satisfactory results are reported after ORIF in children, but the heterogeneity and low level of evidence preclude firm recommendations on the optimal approach.

## 1. Introduction

Paediatric pelvic fractures account for a small proportion (approximately 0.2–2%) of fractures in children and are typically the result of high-energy trauma such as road-traffic accidents or falls from height, frequently associated with visceral, neurological impairment, and head or neck trauma injuries [1,2,3,4]. Hemodynamic instability is less common than in adults but may occur, and management requires a multidisciplinary approach in specialised centres [1,2,3,4].

Acetabular involvement is even rarer, representing a small subset of paediatric pelvic fractures, but is of particular concern because disruption of the developing acetabulum can lead to long-term sequelae such as post-traumatic dysplasia, subluxation, and early osteoarthritis [2,5,6,7,8,9,10]. In children, pelvic and acetabular fractures are predominantly managed non-operatively with protected weight bearing and close follow-up [1,2,3,5]. Surgical treatment is usually reserved for unstable pelvic ring injuries and acetabular fractures with >2 mm articular displacement, intra-articular fragments, or incongruent reduction, applying criteria similar to those used in adults once the triradiate cartilage is closed [1,2,3,4,5,8,10,11].

In younger children with open triradiate cartilage, acetabular fractures pose specific challenges. Injuries to the triradiate cartilage may result in growth disturbance, secondary acetabular dysplasia, and hip subluxation, even after apparently adequate treatment [6,7,8]. Conventional radiographs and Computed Tomography (CT) imaging could underestimate cartilaginous injuries due to incomplete ossification, and Magnetic Resonance Imaging (MRI) is often required to assess the physis (proximal femoral and triradiate cartilage) and associated intra-articular lesions [6,7,10]. Internal fixation must therefore aim not only to restore joint congruency but also to minimise iatrogenic damage to the growth plate.

The evidence base for operative management of paediatric acetabular fractures consists mainly of retrospective series and isolated case reports. Heeg et al. reported 29 acetabular fractures in children and adolescents, of which 14 were treated with open reduction and internal fixation, with long-term outcomes closely related to the quality of reduction [8]. Von Heyden et al., using data from the German Pelvic Trauma Registry, confirmed that acetabular fractures in children are frequently associated with high-energy trauma and concomitant injuries, and that only a minority require operative management [9]. More recent series have described internal fixation using plates and screws in adolescents [10,11], triradiate cartilage plating in a 14-year-old boy [12], lag-screw fixation of posterior wall fractures [13,14,15,16], and novel techniques such as titanium elastic nails [17,18,19].

Although intrapelvic approaches are widely described in adults, paediatric data remain sparse, and the indications, feasibility, and outcomes in skeletally immature patients are not well characterised.

For adult acetabular fractures involving the anterior column and quadrilateral surface, the modified Stoppa (intrapelvic) approach has gained popularity because it offers direct access to the inner pelvis, allows infrapectineal buttress plating, and reduces the need for extensive soft-tissue dissection compared with the classic ilioinguinal approach. Its application in skeletally immature patients, however, has been very rarely reported, with only isolated paediatric cases in the literature [4,5].

The primary aim of the present study is to describe in detail the management and mid-term outcome of an 11-year-old girl with a transverse acetabular fracture and triradiate cartilage disruption treated with ORIF using a modified Stoppa approach and infrapectineal plating. The secondary aim is to provide a systematic review of the literature on internal fixation of paediatric acetabular fractures, with particular emphasis on surgical approaches, fixation methods, and clinical outcomes.

## 2. Materials and Methods

### 2.1. Study Design

**Figure 1:**
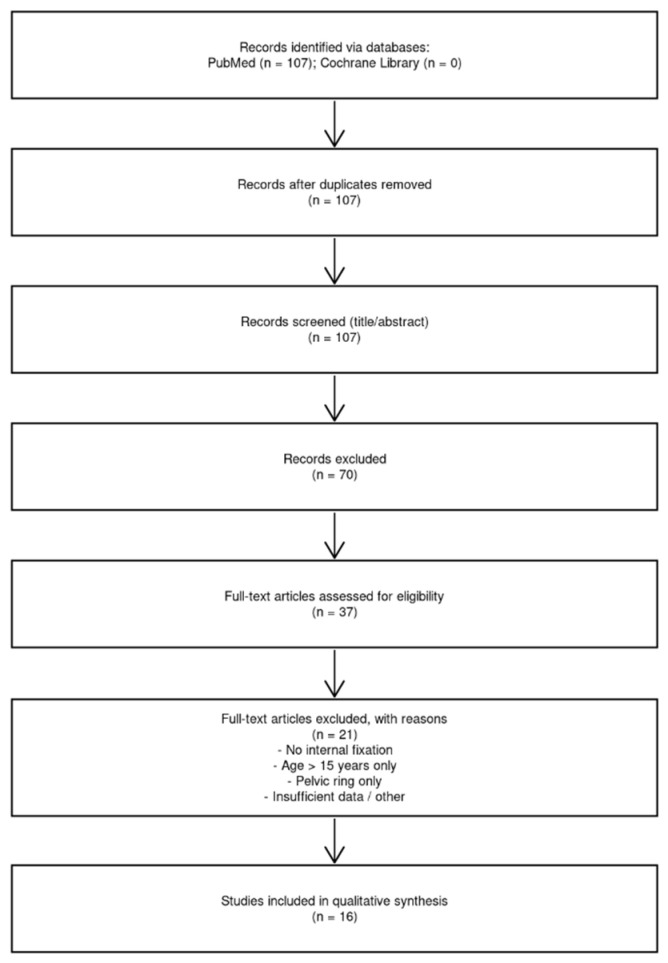
PRISMA 2020 flow diagram illustrates the selection of studies for the systematic review. A total of 107 records were identified in PubMed and none in the Cochrane Library; after removal of duplicates and screening of titles and abstracts, 37 full-text articles on paediatric acetabular trauma were assessed for eligibility, of which 16 met the inclusion criteria and were included in the qualitative synthesis. n = number of articles.

We combined a single-patient case report with a systematic review of the literature on paediatric acetabular fractures treated with internal fixation. The reporting of the review was guided by the PRISMA 2020 statement [20] (Figure 1).

### 2.2. Case Report

An 11-year-old girl, previously healthy and without comorbidities, was transferred to our department (Orthopaedic paediatric unit of Clinica Ortopedica dell’Adulto e Pediatrica, Azienda Ospedaliera “Ospedali Riuniti Ancona Umberto I-G.M. Lancisi-G. Salesi”) after an initial admission to the emergency department of a secondary hospital due to a high-energy road-traffic accident in August 2020. On arrival, she presented with moderate impairment of consciousness and pain in the right lower limb and pelvis.

**Figure 2:**
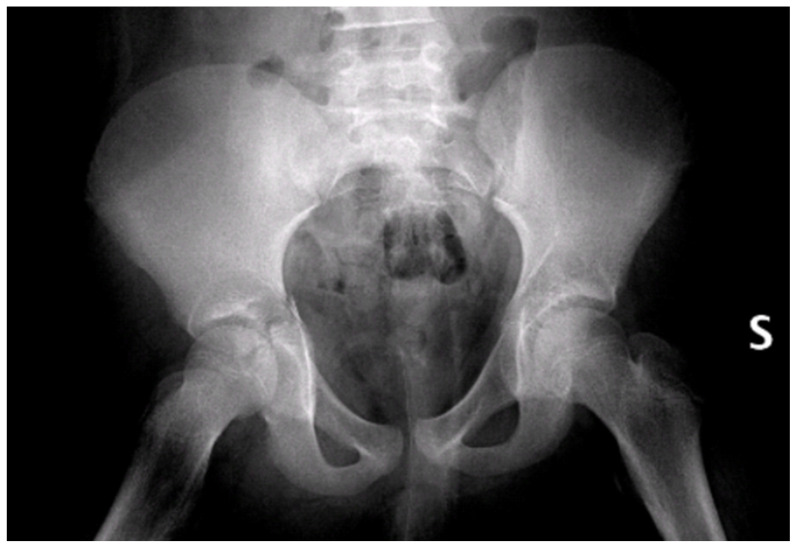
Pelvic X-rays at admission time. S = left side.

**Figure 3:**
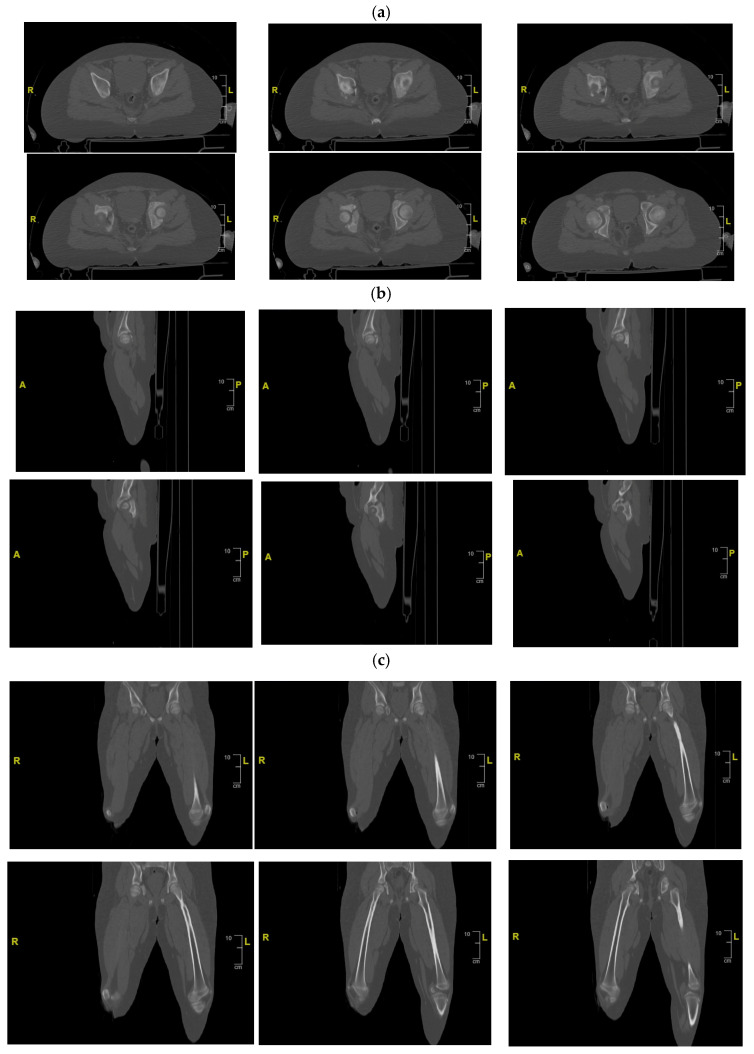
CT of the pelvis showing the right acetabular fracture. (**a**) Axial view; (**b**) sagittal view; (**c**) coronal view. R = right; L = left; A = anterior; P = posterior.

Initial radiographs (Figure 2) and CT imaging revealed an open fracture of the right tibia with involvement of the tibial plateau (Schatzker IV) and a vertical fracture line extending below the tibial tuberosity; a transverse fracture of the right acetabulum with disarticulation of the triradiate cartilage (Figure 3); a suspected fracture at the level of the left lesser trochanter; bilateral pulmonary contusions (segments of the upper and lower lobes); a thin parafalcine and paratentorial intracranial haemorrhage without mass effect; no craniofacial or cervical spine fractures; no solid-organ injury in the abdomen; a small simple cyst in the left kidney; and developmental changes in thoracic vertebral bodies (D6–D8) without acute traumatic lesions. The patient was haemodynamically stable after initial resuscitation. Neurovascular examination of the lower limbs was normal at presentation.

Given the open tibial plateau fracture (Gustilo II), priority was given to the urgent debridement of the right tibia. Broad-spectrum intravenous antibiotic therapy was initiated in the emergency department. On the day of admission, the patient underwent irrigation and debridement of the open tibial fracture, with application of an external fixator spanning the knee (damage-control fixation). Postoperatively, she was monitored in a paediatric intensive care unit.

Definitive management of the acetabular fracture was deferred until the patient’s general condition was optimised and soft-tissue status allowed surgery. A CT scan with multiplanar and 3D reconstructions of the pelvis confirmed a transverse acetabular fracture involving the triradiate cartilage on the right side, with displacement of the quadrilateral surface and medialisation of the femoral head relative to the hemipelvis.

In consideration of the nature of our patient’s fracture, no alternative approach was considered other than the anterior approach, or, in our case, the modified Stoppa approach. This approach enabled the surgeon to exercise control over the fracture at its most critical point and achieve anatomical and stable reduction with significant soft-tissue preservation. The only alternative would have been to use a double approach (anterior and posterior) to treat the fracture. However, this would have had an unacceptable impact from a surgical point of view, particularly when treating a paediatric patient, for whom the main objective must be to minimise invasiveness as much as possible, even when treating soft tissues.

Ten days after the initial damage-control procedure, definitive treatment with ORIF of the acetabular fracture was performed. This was undertaken once both systemic and local conditions were deemed suitable, also due to the presence of the tibial plateau fracture. The timing of the treatment was chosen to allow physiologic stabilisation and to avoid drug overload and overstimulation of the patient’s body.

The patient was positioned supine on a radiolucent table under general anaesthesia. A senior pelvic trauma surgeon performed a modified Stoppa approach. A transverse Pfannenstiel incision of approximately 10 cm was made. After dissection through the subcutaneous tissues, the rectus sheath was split and the rectus abdominis insertion was released from the pubis. The transversalis fascia was incised superior to the symphysis, and the retropubic space of Retzius was entered.

The round ligament in this female patient was identified and protected. The bladder was carefully retracted laterally and superiorly using blunt retractors (PRO Pelvis and Acetabulum System, Stryker, GmbH, Bohnackerweg 1 2545 Selzach, Switzerland). The obturator nerve and vessels, as well as the femoral neurovascular bundle, were identified and protected, and the corona mortis was identified and ligated throughout the procedure. The surgeon stood on the contralateral side of the injured acetabulum.

Subperiosteal dissection along the inner pelvis exposed the pelvic brim, anterior column, and quadrilateral surface. The transverse acetabular fracture and separation of the triradiate cartilage were visualised. Fracture reduction was achieved using a combination of pointed reduction clamps, ball-spiked pushers, and provisional K-wires (PRO Pelvis and Acetabulum System, Stryker, GmbH, Bohnackerweg 1 2545 Selzach, Switzerland) under fluoroscopic guidance (anteroposterior, iliac, and obturator oblique views) to restore the contour of the quadrilateral surface and ensure congruent reduction in the femoral head within the acetabulum.

**Figure 4:**
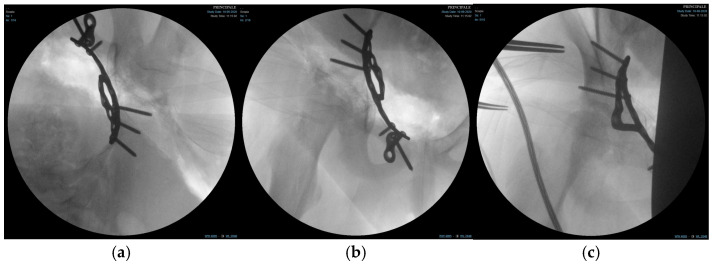
Fluoroscopic images of the surgical treatment of fracture of right acetabulum. (**a**) Iliac projection; (**b**) obturator view; (**c**) anteroposterior view.

Once an anatomical reduction was obtained, a 3D pre-contoured infrapectineal plate (PRO Pelvis and Acetabulum System, Stryker, GmbH, Bohnackerweg 1 2545 Selzach, Switzerland) was applied along the pelvic brim and quadrilateral surface to provide buttress support. The plate was fixed with multiple cortical screws placed in the supra-acetabular corridor and pubic ramus, avoiding penetration of the joint and the triradiate cartilage. The stability of fixation and joint congruency were confirmed fluoroscopically. (Figure 4) Wounds were irrigated and closed in layers over a drain.

Postoperatively, the patient was maintained non-weight bearing on the right lower limb for 4 weeks. Thromboprophylaxis and analgesia were administered according to institutional paediatric protocols; the patient was in the early stages of pubertal development. The external fixator on the tibia was left in situ.

From 4 weeks onwards, progressive weight bearing on the right lower limb was allowed as tolerated, with the external fixator still in place. Clinical and radiographic evaluations (pelvis and right lower limb) were performed immediately postoperatively and at 4, 8, and 12 weeks after acetabular ORIF. The external fixator was removed at 8 weeks once consolidation of the tibial plateau fracture was confirmed radiographically.

**Figure 5:**
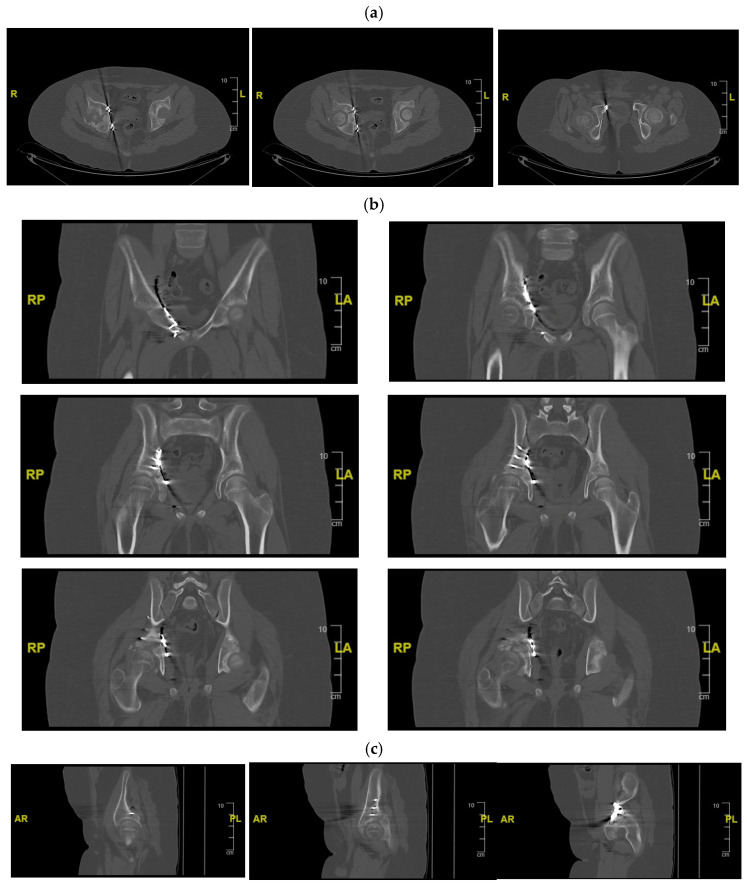
CT of the pelvis showing the synthesis. (**a**) Axial view; (**b**) sagittal view; (**c**) coronal view. R = right; L = left; A = anterior; P = posterior.

**Figure 6:**
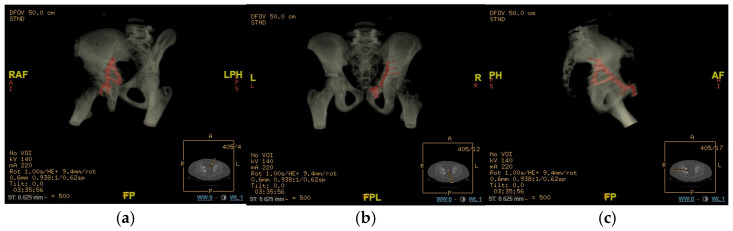
CT render of the plating area of the right acetabulum at 1 month follow-up. (**a**) Oblique view; (**b**) anteroposterior view; (**c**) lateral view. R = right; L = left; A = anterior; P = posterior; H = Head; F = Foot.

**Figure 7:**
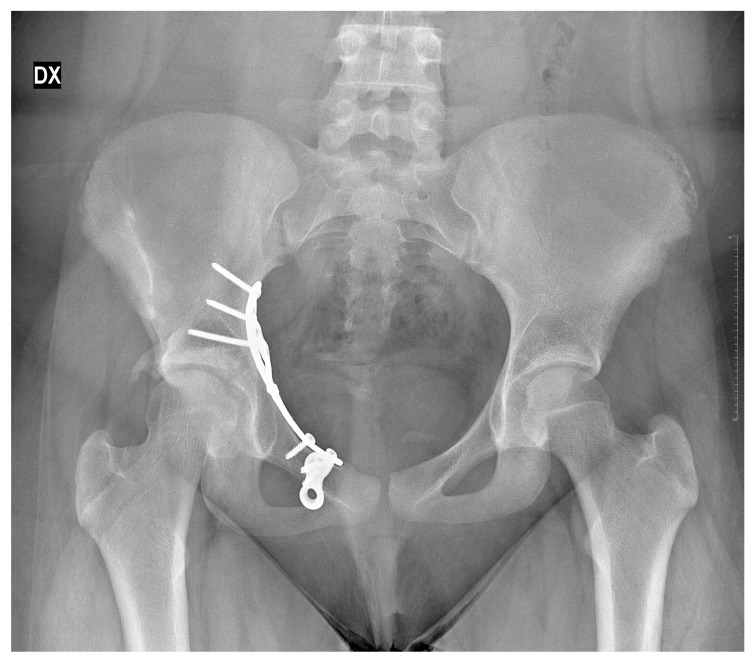
X-rays follow-up at 30 months by the surgical treatment. DX = Right.

Subsequent clinical and radiographic follow-up visits were conducted at approximately 3 months, 6 months, and 4.5 years after definitive fixation hip (last X-ray was performed at 30 months follow-up). After 30 months, no further X-ray examinations were performed in order to avoid exposing the patient to unnecessary radiation for diagnostic or therapeutic purposes (Figure 5, Figure 6 and Figure 7).

Ethical review and approval were waived for this study, as it is a single-patient case report. In this case, according to the policy of our institution, we do not require formal ethical committee approval if informed consent has been obtained. The study was conducted in accordance with the Declaration of Helsinki. Informed consent was obtained from the subject involved in the study and their parents for the use of data relating to the case for research purposes or publication in an anonymous manner. For the review of the literature, no innovative data were being collected, no new databases were being produced, no data analysis was being carried out beyond what is already present and published in the literature. Informed consent is not required.

### 2.3. Systematic Literature Search

A systematic search of PubMed/MEDLINE (via the NIH interface) and the Cochrane Library was performed. The following search terms and combinations were used: “paediatric acetabular osteosynthesis”, “paediatric acetabular internal fixation”, “paediatric ORIF acetabular fracture”, and “paediatric acetabular plating”. Boolean operators and MeSH terms were applied where appropriate. No language or publication date restrictions were imposed.

The search yielded 107 records in PubMed; no relevant records were retrieved in the Cochrane Library. After the removal of duplicates, titles and abstracts were screened to identify studies focusing on paediatric acetabular trauma. Thirty-seven articles dealing with paediatric acetabular fractures were retained for full-text assessment. Consistent with the PRISMA approach, selection steps were recorded, and reasons for exclusion were noted [20].

### 2.4. Eligibility Criteria

Inclusion criteria for the review were

- Original clinical studies (retrospective or prospective), case series, or case reports;
- Patients aged 0–15 years at the time of injury;
- Radiologically confirmed acetabular fracture;
- Surgical treatment of the acetabular fracture with internal fixation (plates, screws, or flexible nails), either as primary or optional treatment;
- Reporting of clinical and/or radiographic outcomes.

Exclusion criteria were

- Studies exclusively addressing pelvic ring fractures without acetabular involvement;
- Adult or mixed series without extractable paediatric data;
- Reviews, conference abstracts without full text, technical notes without patient data, and biomechanical or cadaveric studies;
- Studies in adolescents ≥ 15 years if paediatric data could not be separated.

After applying these criteria, 16 articles were identified that reported surgical treatment with internal fixation of acetabular fractures in skeletally immature patients and were included in the qualitative synthesis.

### 2.5. Data Extraction and Synthesis

From each eligible study, the following data were extracted when available: study design, number of paediatric patients with acetabular fractures, age range, fracture pattern (e.g., Judet–Letournel classification; triradiate cartilage injury), associated injuries, surgical approach, type of fixation, time to surgery, postoperative protocol, follow-up duration, clinical and radiographic outcomes, and complications (e.g., avascular necrosis, growth disturbance, heterotopic ossification, nerve injury).

Given the heterogeneity of study designs and outcome measures, a qualitative narrative synthesis was undertaken rather than a formal meta-analysis.

A formal risk-of-bias tool (e.g., RoB2/ROBINS-I) was not applied because the included evidence base consisted predominantly of case reports and small retrospective case series, for which these instruments are not well suited and would provide limited discriminatory value. Instead, we extracted study design and key reporting elements and interpreted findings descriptively. The absence of a formal risk-of-bias assessment is acknowledged as a limitation and reflects the low level and heterogeneity of the available evidence.

Given the predominance of case reports/series, a simplified quality assessment approach was used: (1) classifying each study by level of evidence based on design, and (2) recording key reporting-quality elements (fracture classification/triradiate cartilage involvement, surgical approach and fixation details, follow-up duration, and complication reporting). Findings were synthesised narratively and interpreted, with greater weight given to studies with clearer methods, longer follow-up, and more complete outcome reporting.

The authors declare that generative artificial intelligence (GenAI) was used in this paper as support to draw tabs and graphics.

## 3. Results

### 3.1. Case Outcome

At four weeks postoperatively, the patient had healed surgical wounds with no signs of infection. Hip range of motion was already near full in flexion and rotation, limited mainly by discomfort and the presence of the tibial external fixator. Radiographs showed maintained reduction in the acetabular fracture and satisfactory alignment of the tibial plateau.

At eight weeks, with radiographic evidence of consolidation of the tibial plateau fracture, the external fixator was removed. The patient reported minimal pain and was able to increase weight bearing with the aid of crutches.

At approximately three months after acetabular ORIF, the patient walked independently with full weight bearing on the right lower limb without pain. Clinical examination revealed

- Full range of motion of the right hip compared with the contralateral side;
- Full range of motion of the right knee and ankle;
- Persistent deficit of active extension of the right great toe, with hypoesthesia over the dorsum of the right foot in the distribution of the superficial peroneal nerve; sensibility in the first webspace and distal pulses were preserved.

An electromyography (EMG) of the right lower limb was requested to characterise the nerve deficit. The patient continued with home-based physiotherapy. Subsequent reviews at two and three months documented progressive improvement in ankle and toe dorsiflexion, with clinical resolution of the superficial peroneal neuropathy over time.

At six-month follow-up, the patient walked normally without pain or limp and without leg-length discrepancy. The right knee range of motion was symmetrical to the contralateral limb. Hip examination showed a very mild loss of flexion compared with the left hip, with otherwise full range in all planes. She had returned to normal daily activities and was cleared to resume sport. Residual weakness of great-toe extension and mild hypoesthesia on the dorsum of the foot persisted but did not interfere with function.

At the most recent follow-up, May 2025 (4.5 years after surgery), the patient was overweight but otherwise well. Clinical examination showed

- Well-healed scars;
- Full, pain-free range of motion of both hips and knees;
- Negative FABER and FADIR tests bilaterally;
- Normal neurovascular status in both lower limbs, with no clinically appreciable motor deficit or sensory loss;
- Normal gait without Trendelenburg sign or limp, and no apparent discrepancy in limb length.

Pelvic radiographs demonstrated a congruent right hip joint (at 30 months of follow-up), maintained reduction in the acetabulum with well-positioned hardware, and no evidence of premature closure of the triradiate cartilage, acetabular dysplasia, coxa magna, avascular necrosis, or degenerative joint changes. The patient was advised to pursue weight reduction and regular physical activity; further reviews were scheduled on an as-needed basis.

### 3.2. Literature Search Results

The authors identified 107 records in PubMed and none in the Cochrane Library. After the removal of duplicates and screening of titles and abstracts, 37 studies focusing on paediatric acetabular trauma were reviewed in full. Sixteen studies met the inclusion criteria by reporting surgical internal fixation of acetabular fractures in patients aged 0–15 years and were included in the qualitative synthesis.

These comprised

- Three retrospective series including both children and adolescents with acetabular fractures, with a subset managed by ORIF [8,9,10,11,18];
- One registry-based cohort study on paediatric acetabular fractures [9];
- Several case reports describing internal fixation using plates or screws [7,12,13,14,15,16];
- One case report describing a novel technique using titanium elastic nails for acetabular fracture stabilisation in a skeletally immature patient [17].

### 3.3. Characteristics of Included Studies

Table 1 summarises the main characteristics and peculiarities of the included studies and the present case. Across the series, acetabular fractures were most often associated with high-energy trauma, predominantly road-traffic accidents [8,9,10,11,12,14,15,17]. Posterior wall fractures, transverse and T-type patterns, and fractures involving the triradiate cartilage were the most frequently reported morphologies [8,9,10,11,12].

The indications for internal fixation were broadly consistent with adult criteria: articular displacement > 2 mm, intra-articular fragments, unstable fracture patterns, and associated hip dislocation or subluxation [1,2,3,4,5,8,9,10,11]. However, the authors emphasised the need to consider the status of the triradiate cartilage and the potential impact of iatrogenic physeal injury when planning fixation [6,7,8,9,10,12,17].

## 4. Discussion

In our work, we collected several typical features of paediatric pelvic and acetabular trauma: a high-energy mechanism (road-traffic accident), associated limb fractures, and concomitant systemic injuries. Large series and reviews have consistently reported that paediatric pelvic girdle injuries are rare and usually reflect major trauma, with a relatively high rate of associated head, thoraco-abdominal, and limb injuries [1,2,3,4,5,9,18]. Early recognition and systematic radiological assessment are essential, as acetabular involvement may be under-diagnosed in the acute setting, particularly in young children with incompletely ossified acetabula [5,6,7].

While most paediatric pelvic and acetabular fractures can be managed non-operatively with closed reduction and protected weight bearing, several authors advocate operative treatment when adult-type displacement or instability criteria are present [1,2,3,4,5,8,9,10,11,12]. In particular, operative indications include

- Articular displacement > 2 mm;
- Intra-articular fragments or incarcerated bone/soft tissue;
- Irreducible or unstable hip dislocations associated with acetabular fractures;
- Transverse, T-type, or both-column fractures with incongruous reduction;
- Markedly displaced triradiate cartilage fractures.

In our patient, the presence of a transverse acetabular fracture with disruption and displacement of the triradiate cartilage and medialisation of the femoral head justified ORIF to restore joint congruency, re-establish acetabular anatomy, and minimise the risk of growth disturbance.

The management of the associated open tibial plateau fracture illustrates the principle of damage-control orthopaedics in paediatric polytrauma. Early external fixation of the tibia allowed stabilisation of the limb, facilitated nursing care, and permitted the safe delay of definitive acetabular surgery until the child’s overall condition was optimised.

When ORIF is indicated, definitive fixation should be undertaken after hemodynamic/physiological stabilisation, and once early pelvic bleeding and soft-tissue swelling have subsided—ideally within the first week (≈3–7 days) and generally within 10 days—while any associated hip fracture–dislocation should be reduced emergently (preferably <6 h) to reduce the risk of AVN and early osteoarthritis [2,4,8,10,18,19,21,22].

In paediatric acetabular fractures requiring surgery, most published series and case reports have used standard adult approaches such as the ilioinguinal, Kocher–Langenbeck, or combined approaches, with fixation using reconstruction plates and screws [8,9,10,11,12,13,14]. Anterior approaches are usually selected for anterior column and triradiate cartilage injuries, while posterior approaches are used for posterior wall and posterior column fractures.

The modified Stoppa approach, first popularised in adult acetabular surgery, provides intrapelvic access to the anterior column, pelvic brim, and quadrilateral surface through a relatively small Pfannenstiel incision. It allows direct buttress plating of the quadrilateral surface with infrapectineal plates and offers a favourable corridor for supra-acetabular screws, often with reduced soft-tissue dissection compared with the classic ilioinguinal approach [4,5]. Although widely adopted in adults, its use in paediatric acetabular fractures has been only sporadically described.

In our case, the modified Stoppa approach was particularly advantageous in treatment the T-shaped acetabular fracture of the paediatric patient because

- It provided direct visualisation of the medially displaced quadrilateral surface and triradiate cartilage region.
- It allowed anatomical reduction in the transverse fracture from the inner pelvis.
- It permitted the application of a pre-contoured infrapectineal plate acting as a buttress to prevent medial migration of the femoral head.
- It avoided extensive dissection of the iliac wing and inguinal region.
- The fracture was treated without using a double approach, which made it less invasive and better preserved the soft tissue.

Given the transverse configuration of the fracture with the involvement of anterior and posterior column, we chose to address the main deformity through an endopelvic pathway, aiming to restore acetabular congruity and reduce the risk of post-traumatic osteoarthritis [1,4,9,13]. A combined anterior–posterior (double) approach could be considered but ultimately avoided because of the greater surgical burden and soft-tissue morbidity in a paediatric patient. In this context, a modified Stoppa (anterior intrapelvic) approach may be reasonable in children when the fracture pattern requires direct visualisation and buttress control of the inner pelvis—particularly in transverse or associated both-column patterns with quadrilateral surface involvement, anterior column displacement, or femoral head medialisation that can be reduced and stabilised from an intrapelvic window [1,4]. Its use is especially attractive when a single approach can adequately manage both the anterior component and the critical endopelvic displacement, provided the surgeon has specific expertise with pelvic/acetabular surgery and familiarity with paediatric anatomical constraints. Careful identification and protection of the corona mortis, obturator neurovascular bundle, and femoral vessels are crucial in this approach, and the smaller pelvic dimensions in children demand meticulous technique and familiarity with paediatric pelvic anatomy.

Conversely, alternative approaches may be preferred when fracture morphology demands direct access to areas not reliably addressed through Stoppa—such as isolated posterior wall/column fractures or patterns with substantial posterior comminution (often better served by a Kocher–Langenbeck approach), high anterior column or iliac wing extension requiring broader anterior exposure (where an ilioinguinal or other anterior approach may be advantageous), or situations in which adequate reduction cannot be achieved safely through an intrapelvic window and a combined approach becomes necessary despite the added invasiveness [7,13]. Finally, minimally displaced fractures may be amenable to nonoperative care in selected cases, but careful follow-up is required given the potential for growth-related sequelae [1,9,17].

The surgeon’s experience with the Stoppa and Stoppa-modified approach should not be underestimated, and it is part of the decision-making process. This factor may limit the application of the modified Stoppa approach even when the fracture pattern would theoretically be amenable, particularly in centres where paediatric acetabular surgery is infrequent and dedicated pelvic expertise is not routinely available [2,3,4]. Conversely, meticulous preoperative planning is essential: careful CT-based assessment, correct classification, and precise definition of fracture morphology are required to identify when an intrapelvic exposure will truly add value (e.g., endopelvic displacement of the quadrilateral surface or femoral head medialisation requiring buttress control). Applying a modified Stoppa approach to an incorrectly characterised fracture risks unnecessary morbidity—potentially subjecting the patient to a more extensive operation when a simpler and less invasive approach could have achieved an equivalent reduction and fixation [1,4,13].

Our fixation strategy using a dedicated 3D pre-contoured infrapectineal plate and screws is in line with the growing trend towards anatomically shaped implants adapted from adult pelvic surgery. In our patient, screws were positioned away from the physeal cartilage and joint, guided by fluoroscopy during the surgery, to avoid direct damage to the growing cartilage or to the joint. As the data in the literature shows, particular care must be taken in skeletally immature patients to avoid penetration of the triradiate cartilage and to respect the remaining growth potential [1,6,10,13].

Across the included series in the literature, good to excellent functional outcomes were generally observed when anatomical reduction and stable internal fixation were achieved [8,9,10,11,12,13]. Heeg et al. reported satisfactory long-term results in most surgically treated patients but noted that residual displacement and associated hip dislocation, neurological injury, or femoral head involvement were associated with worse prognosis [8]. Southam et al. found that 86% of their paediatric and adolescent patients treated with ORIF had favourable functional outcomes at a mean of more than five years, but two cases with delayed reduction in fracture-dislocations developed avascular necrosis and early osteoarthritis [12].

Case reports focusing on triradiate cartilage injuries treated with plating have demonstrated radiographic symmetry of the acetabula and absence of dysplasia at medium-term follow-up when anatomical reduction was obtained [13]. Conversely, cases presenting with complete transphyseal separation of the femoral head or severe physeal damage often progress to avascular necrosis and joint degeneration despite early surgical intervention [17,18,19]. This underscores the importance of prompt recognition of physeal injuries and realistic counselling regarding prognosis.

Although we avoided transphyseal screw placement, we acknowledge the theoretical concern that a construct spanning an active physis might act as a “tether” analogous to temporary hemiepiphysiodesis (tension-band/eight-plate) used for guided growth in limb deformity correction [23]. With the aim to treat the fracture and stabilising it, our infrapectineal fixation was designed as a buttress to restore acetabular congruency and stabilise the quadrilateral surface/columns, with extra-physeal screw placement under fluoroscopic guidance and without direct triradiate cartilage violation. Triradiate cartilage closure is staged in early adolescence and is typically complete by approximately 13–14 years in females (with variability); CT-based series report closure around the mid-teens [24]. At early follow-up in our patient, fracture union was achieved, and the triradiate cartilage was effectively fused, making routine implant removal a relative rather than absolute indication. Moreover, traumatic triradiate cartilage injuries are themselves associated with bone bridge formation and premature closure, which can drive growth disturbance independently of fixation [25]. Therefore, in an asymptomatic transitional-age patient without radiographic dysplasia or clinical impairment, we elected to retain the plate.

In our case, at 4.5 years after surgery, the patient remained asymptomatic with full range of motion and a radiographically congruent hip (last X-ray was performed at 30 months follow-up), and no evidence of acetabular dysplasia, growth disturbance or avascular necrosis. Although longer radiographical follow-up into skeletal maturity is desirable, these findings suggest that stable anatomical reconstruction using a modified Stoppa approach and infrapectineal plating can provide durable mid-term results in selected skeletally immature patients.

In the literature, reported complications of acetabular ORIF in children include infection, heterotopic ossification, nerve injury (particularly sciatic and femoral nerve), avascular necrosis, post-traumatic arthritis, and growth disturbance [8,9,10,11,12]. In our patient, a transient neuropathy of the superficial peroneal nerve, likely related to the tibial injury and external fixation rather than the acetabular surgery, improved over time and did not result in permanent functional limitation.

The strengths of this work include the detailed clinical and imaging documentation, a step-by-step description of the modified Stoppa technique being performed in a skeletally immature patient with triradiate cartilage involvement, and mid-term follow-up after a rare paediatric transverse acetabular fracture. The systematic review focuses specifically on open reduction and internal fixation (ORIF) in skeletally immature patients, summarising the heterogeneous strategies currently reported.

However, several limitations must be acknowledged. Firstly, this is a single-case report, and the described outcome and technical feasibility cannot be extended indiscriminately to all the acetabular fracture patterns, particularly given the need for specialised pelvic/acetabular expertise in treating surgically this type of fracture in children. Secondly, the review is constrained by the heterogeneity and predominantly low level of evidence in the literature (mainly case reports/retrospective series) with inconsistent classification, treatment protocols, and outcome reporting. High-quality comparative studies are largely absent, precluding robust recommendations and limiting synthesis to a descriptive, hypothesis-generating level. These constraints support the need for multicentre collaborative registries with standardised data capture and outcome measures to enable meaningful comparisons and refine indications for surgical approaches in paediatric acetabular fractures. At least, we did not perform a formal risk-of-bias assessment or meta-analysis because of the limited and heterogeneous data.

## 5. Conclusions

Acetabular fractures in children are rare but potentially devastating injuries, typically resulting from high-energy trauma. Although most can be managed non-operatively, fractures with clinically relevant displacement, intra-articular incongruity, or triradiate cartilage disruption may require open reduction and internal fixation to restore joint congruency and reduce the risk of late sequelae.

In our work, we have described the application of a modified Stoppa (anterior intrapelvic) approach—well established in adult acetabular surgery—as a feasible and safe surgical pathway in a selected skeletally immature patient with a transverse acetabular fracture involving the triradiate cartilage. In our case, the approach enabled the anatomical reconstruction of the anterior column and quadrilateral surface with infrapectineal plating and was associated with excellent clinical outcomes at 4.5-year follow-up. These observations should be interpreted as demonstrating technical applicability in an appropriate fracture pattern, rather than superiority over alternative approaches.

The available literature on paediatric acetabular osteosynthesis remains limited and consists mainly of case reports and small retrospective series, precluding definitive guidance on the optimal surgical approach or fixation construct. Accordingly, management should be individualised based on fracture morphology, skeletal maturity, associated injuries, and surgeon expertise. Prospective multicentre registries and long-term follow-up studies are needed to better define indications and comparative outcomes of operative strategies—including intrapelvic approaches such as modified Stoppa—within this vulnerable population.

## Figures and Tables

**Table 1:** Summary of paediatric acetabular fractures treated with internal fixation identified in the literature and comparison with the present case.

First Author, Year	Study Type/n (Acetabular ORIF in ≤15 Years)	Age Range (Years)	Main Fracture Pattern(s)	Surgical Approach(es)	Fixation Method(s)	Follow-Up (Approx.)	Main Outcomes and Peculiarities
Heeg, 2000 [8]	Retrospective series; 14 ORIF among 29 paediatric acetabular fractures	2–16	Variety of Judet–Letournel patterns; frequent associated hip dislocation and neurological injury	Mainly ilioinguinal and Kocher–Langenbeck	Plates and screws; lag screws	Mean ≈ 14 years	Quality of reduction strongly correlated with long-term clinical outcome; growth disturbance and post-traumatic arthritis in cases with residual incongruity.
von Heyden, 2012 [9]	Registry-based cohort (German Pelvic Trauma Registry); subset treated operatively	Mostly < 16	Mixed pelvic and acetabular fractures; high rate of associated injuries	Standard adult approaches (not always specified)	Internal fixation when adult-type indications met	Variable	Acetabular fractures in children are rare, high-energy injuries; only a minority require ORIF; outcomes acceptable when reduction is anatomical.
de Ridder, 2019 [4]	Retrospective series of paediatric pelvic and acetabular fractures requiring surgery	Not specified	Unstable pelvic ring and acetabular fractures, including transverse and posterior wall	Ilioinguinal, Kocher–Langenbeck, combined approaches	Plates and screws	Medium-term	ORIF of paediatric pelvic and acetabular fractures is safe and effective when performed in specialised centres; adult principles applicable in older children.
Tomaszewski, 2021 [10]	Retrospective series; 6 surgically treated acetabular fractures among 9 patients	12–16.5	Mostly transverse/posterior column injuries; some triradiate cartilage involvement	Kocher–Langenbeck and ilioinguinal	Plates and screws; lag screws	Mean 6.2 years	Acetabular fractures constitute ≈ 1–4.6% of paediatric fractures; MRI recommended to evaluate triradiate cartilage; good outcomes when anatomy restored.
Southam, 2022 [11]	Retrospective cohort; 34 ORIF in patients < 18 y (21 with follow-up)	<18	Displaced fractures meeting adult indications; many with hip dislocation	Adult standard approaches (often posterior, sometimes anterior/combined)	Plate and screw constructs	Mean 5 years	86% had favourable functional outcomes; poor results mainly related to delayed reduction and development of avascular necrosis and osteoarthritis.
Tomaszewski, 2011 [19]	Retrospective series of operative pelvic fractures in children	Mostly > 8	Unstable pelvic ring fractures; some acetabular involvement	Various	Plates, screws, external fixation	Up to 12 months	Emphasised principles of stable fixation and early rehabilitation; highlighted technical challenges in osteosynthesis in children.
Rubel, 2002 [7]	Case series; 2 children	Not specified	Posterior wall fracture with traumatic hip dislocation	Posterior approach	Screw fixation of posterior wall; guided by MRI findings	Not specified (short- to mid-term)	MRI shown to be superior to radiographs/CT in detecting posterior wall injury and guiding decision for ORIF in children.
Fitze, 2008 [14]	Case report; 1 patient	13	Posterior wall (avulsion-type) fracture after minor trauma	Posterior approach	Three lag screws	2 years	Full recovery and return to sport; highlights that even low-energy trauma may cause acetabular fracture in adolescents.
Spina, 2019 [12]	Case report; 1 patient	14	Displaced triradiate cartilage fracture (Salter–Harris type I)	Anterior approach	Matta plate across triradiate cartilage	2 years	Symmetrical acetabular development and complete fusion of triradiate cartilage; advocates plating in markedly displaced triradiate injuries.
Palencia, 2016 [16]	Case report; 1 patient	12	Complete transphyseal separation of femoral head with minimally displaced anterior column fracture	Anterior approach	Cannulated screws for epiphysis (acetabular fracture treated conservatively)	6 months+	Developed femoral head osteonecrosis despite early reduction; illustrates poor prognosis of transphyseal hip injuries associated with acetabular fracture.
Mohamed, 2019 [16]	Case report; 1 patient	13	Bilateral acetabular fracture-dislocations after epileptic seizures	Pfannenstiel with intrapelvic exposure	Supra-pectineal plates	Short- to mid-term	Demonstrated feasibility of bilateral acetabular ORIF via Pfannenstiel-based approach in a paediatric patient.
Razak, 2021 [18]	Case report; 1 patient	8	Anterior column posterior hemitransverse acetabular fracture	Minimally invasive percutaneous corridors	Three titanium elastic nails (TENS)	Short-term	Novel intramedullary fixation strategy aiming to reduce physeal injury; good early outcome; emphasises narrow safe corridors in children.
Liu, 2023 [15]	Case report; 1 patient	13	Isolated posterior wall acetabular fracture	Kocher-Langenbeck	Three lag screws	Short-term	Favourable early outcome of complex paediatric acetabular ORIF; underscores importance of anatomical reconstruction.
Slongo, 2013 [6]	Narrative review including operative cases	Not specified	Acetabular fractures including triradiate injuries	Various	Plates and screws when indicated	Not applicable	Highlights anatomical peculiarities in young children; recommends liberal MRI use and expert surgical teams.
Present case	Case report; 1 patient	11	Transverse acetabular fracture with triradiate cartilage disruption; associated open Schatzker IV tibial plateau fracture	Modified Stoppa (intrapelvic) approach	Pre-contoured infrapectineal plate and screws; staged with external fixation for tibia	4.5 years	Excellent clinical and radiographic outcome; no evidence of dysplasia or AVN; demonstrates feasibility of modified Stoppa approach and infrapectineal plating in a skeletally immature patient.

## Data Availability

No new datasets were created or analysed in this study. Data sharing is not applicable to this article.
